# Supplementary material for: RASAL1 and ROS1 Gene Variants in Hereditary Breast Cancer
Source: Cancers (Basel). 2020 Sep 7;12(9):2539. doi: 10.3390/cancers12092539 (PMC7563829; doi:10.3390/cancers12092539)
Supplement: Supplementary file 1 [file cancers-12-02539-s001.pdf]

Supplementary Materials: RASAL1 and ROS1 Gene Variants in Hereditary Breast Cancer

Federica Isidori, Isotta Bozzarelli, Simona Ferrari, Lea Godino, Giovanni Innella, Daniela Turchetti and Elena Bonora

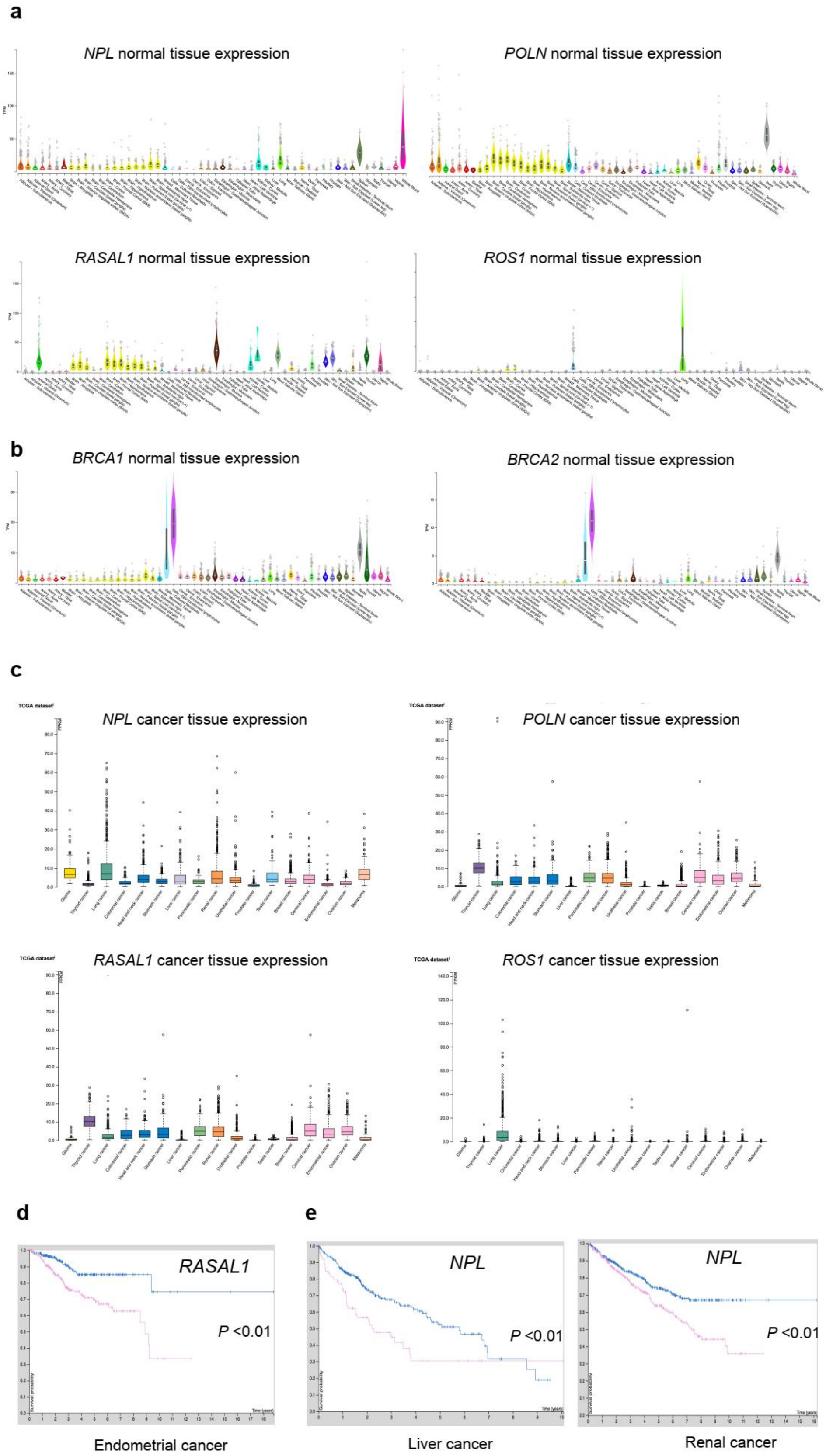

Figure S1. Gene expression in human tissues. (**a**) Expression levels shown for *NPL*, *POLN*, *RASAL1* and *ROS1* in human normal tissues (GTEx portal). (**b**) Expression levels shown for *BRCA1* and *BRCA2* in human normal tissues (GTEx portal). (**c**) Expression levels shown for *NPL*, *POLN*, *RASAL1* and *ROS1* in cancer tissues (Human Protein Atlas database). (**d**) Kaplan-Meier survival curve for *RASAL1* in endometrial cancer, showing that overexpression of *RASAL1* (violet line) was significantly

associated to poor outcome, compared to samples not showing *RASAL1* overexpression (blue line). (**e**) Kaplan-Meier survival curves for *NPL* in liver and renal cancers, showing that overexpression of *NPL* (violet line) was significantly associated to poor outcome, compared to samples not showing *NPL* overexpression (blue line). Data extracted from Human Protein Atlas.

Table S1. Clinical and familial features of the BC patients included in the target NGS screening.

**Features of Patients**		
Mean Age at BC Diagnosis		38.73 ± 8.93 (years)
Bilateral BC		11 (8.4%)
Number of first-degree relatives with BC	0	75 (57.3%)
	1	46 (35.1%)
	2	8 (6.1%)
	3	2 (1.5%)
Number of first-degree relatives with Ovarian Cancer	0	126 (96.2%)
	1	5 (3.8%)
Criterion for BRCA testing according to RER protocol	Hereditary/familial site-specific BC	48 (36.6%)
	Hereditary/familial Breast/Ovarian Cancer	8 (6.1%)
	Early-onset BC (<36y)	51 (38.9%)
	Male BC	5 (3.8%)
	Triple-negative BC <60y	19 (14.5%)

Table S2. Novel/rare variants in *POLN, NPL* genes detected through target NGS analysis.

**cDNA Position** [a]	**Functional Effect**	**MAF BC (*n* = 131)**	**MAF gnomAD (European Non-Finnish)**
POLN c.1554 + 6G > C (rs189543362)	Splice region	0.38 1/262	0.00004651 6/129010 (*p* = 0.0141) [b]
POLN c.2308 + 1G > A (rs143143974)	Altering canonical splice donor	0.38 1/262	0.0004028 52/129110 (*p* = 0.1019) [b]
NPL c.*811C > T (rs148028376)	3′UTR	0.38 1/262	0.00002838 2/70468 (*p* = 0.0111) [b]

(a) *POLN* NM_181808, *NPL* NM_030769.2. (b) *p* values calculated using the Fisher exact test, considering the minor allele frequencies reported in gnomAD, accessed on 19 May 2020.

Table S3. Features of patients according to *ROS1* and *RASAL1* test results.

Patient Features		*ROS1* Variant (n = 22)	*RASAL1* Variant (n = 5)	No Variants (n = 104)	p
Male proband		1 (4.5%)	0	3 (2.9%)	0.608 [a]
Mean age (years) at BC diagnosis		41.36 ± 11.31	36.20 ± 5.45	38.29 ± 8.452	0.358 [b]
Bilateral BC		4 (18.2%)	0	7 (6.7%)	0.183 [a]
Personal history of OC		0	0	3 (2.9%)	1.000 [a]
Family history of BC in first-degree relatives		12 (54.5%)	0	44 (42.3%)	0.075 [a]
Family history of male BC		0	1 (20%)	3 (2.9%)	0.160 [a]
Criterion for BRCA testing according to RER protocol	Hereditary/familial site-specific BC	2 (9.1%)	0	6 (5.8%)	0.880 [a]
	Hereditary/familial Breast/Ovarian Cancer	10 (45.5%)	1 (20.0%)	37 (35.6%)	
	Early-onset BC (<36 y)	7 (31.8%)	3 (60.0%)	41 (39.4%)	
	Male BC	1 (4.5%)	0	4 (3.8%)	
	Triple-negative BC <60 y	2 (9.1%)	1 (20.0%)	16 (15.4%)	

[a] Fisher's exact test. [b] Kruskal–Wallis test.

Table S4. Mutation frequencies of cases in *BRCA1*, *BRCA2, ROS1*, *RASAL1* in breast cancer cases, as reported in COSMIC.

Gene	Mutated Samples in COSMIC	Frequency (%)	*p* Value (χ^2-test)
BRCA1	229/8090	2.83	--
BRCA2	306/7532	4.06	--
ROS1	217/4792	4.53	*<0.00001 (ROS1* vs. *BRCA1)*<br>0.211206 (*ROS1* vs. *BRCA2*)
RASAL1	33/2583	1.28	<0.00001 *(RASAL1* vs. *BRCA1)*<br><0.00001 (*RASAL1* vs. *BRCA2*)

© 2020 by the authors. Licensee MDPI, Basel, Switzerland. This article is an open access article distributed under the terms and conditions of the Creative Commons Attribution (CC BY) license (http://creativecommons.org/licenses/by/4.0/).
